# Enhancing adolescents’ exercise motivation management with generative AI anthropomorphism: a chain-mediated model of technology acceptance and self-efficacy

**DOI:** 10.3389/fpsyg.2025.1662331

**Published:** 2025-11-19

**Authors:** Kaiyuan Wang, Hongxin Li, Guo Yu, Gang Li, Yu Song

**Affiliations:** 1 School of Economics and Management, Shanghai University of Sport, Shanghai, China; 2 School of Sport Exercise and Health Sciences, Loughborough University, Loughborough, United Kingdom; 3 Physical Education College, Shandong University of Finance and Economics, Jinan, China

**Keywords:** AI anthropomorphism, technology acceptance, self-efficacy, exercise motivation, adolescents, multi-group SEM

## Abstract

**Introduction:**

In the era of human-machine integration, digital technology highlights its important enabling role in the development of youth sports. The objective of this study is to examine the mechanism through which AI Anthropomorphism is associated with exercise motivation among adolescents.

**Methods:**

The data were derived from a sample of 1,018 adolescents aged 6–18 across the country. The AI anthropomorphism scale, the Generative artificial intelligence acceptance (GAIA), the General Self-Efficacy Scale (GSES), and the Physical Activity Motivation Scale (MPAM-R) were used to assess AI anthropomorphism, technology acceptance, self-efficacy and exercise motivation.

**Results:**

This study found that AI anthropomorphism is significantly associated with adolescents’ motivation for physical activity. Further analysis reveals that technology acceptance and self-efficacy independently serve as mediators and chain mediators, respectively, in this relationship, clarifying the underlying psychological mechanisms.

**Conclusion:**

This study elucidates the mechanism by which AI anthropomorphism is associated with adolescents’ motivation for exercise motivation, offering theoretical support for understanding such behavior and practical guidance for implementation.

## 1 Introduction

The development of artificial intelligence (AI) demonstrates a trend of rapid growth. According to McKinsey and Company, AI contributes between $2.6 and $4 trillion annually to the global economy. (McKinsey and Company, 2024a,b). Among the various technical directions of AI, anthropomorphic technology has attracted wide attention for its ability to improve the interaction between humans and machines. Although AI anthropomorphism has been widely focus, research in the youth sports field remains urgently needed. From a practical perspective, insufficient physical activity among children and adolescents has become a global issue (Liu et al., 2023), necessitating the exploration of more effective measures to enhance youth physical activity levels.

Nowadays, AI-related research in the sports mainly focuses on injury risk prediction (Musat et al., 2024), athletic performance enhancement (Puce et al., 2024), and equipment material optimization (Li Y. et al., 2024). However, existing research has primarily focused on adults, overlooking the greater alignment between AI anthropomorphism and the psychological development needs of adolescents (Chew, 2022). Moreover, existing research has demonstrated that the impact of educational chatbots on university students is more pronounced compared to their influence on secondary school students (Wu and Yu, 2024). In fact, AI anthropomorphism demonstrates a high degree of compatibility with fulfilling adolescents’ physical activity needs from a physiological perspective. Specifically, young people show delayed development of the prefrontal cortex, increased activity in the amygdala (Foulkes and Blakemore, 2018) and increased sensitivity to stress-related systems compared to adults (Romeo), leading them to place greater emphasis on social acceptance and social evaluation. Therefore, AI anthropomorphism is more closely related to adolescents’ physical exercise.

Current AI research in youth sport shows some theoretical gaps. First, the independent effect of anthropomorphic AI traits as a predictor variable has not been adequately addressed in the current studies. Existing research indicates that technology acceptance is improved by enjoyment (Sohn and Kwon, 2020), system quality (Won et al., 2023), and perceived usefulness (Wang et al., 2022); self-efficacy is associated with multiple factors including role models (Kleppang et al., 2023), perceived environment (Wu et al., 2025), peer support (Lin et al., 2024), and technological factors (Pan, 2020). However, the relationship between AI anthropomorphism and these variables remains unclear. Second, the formation of exercise motivation is a complex process involving the sequential interaction of multiple factors. However, existing studies have explored the instrumental (Saraç et al., 2025) and affective (Lee and Lin, 2023) aspects of AI anthropomorphism separately, which is not facilitate a comprehensive and systematic exposition of the formation of exercise motivation. The chain mediation model is well-suited to elucidate this complex mechanism.

In order to fill this research gap, the study takes into account both technological and psychological factors. By comparing similar variables and frameworks across UTAUT, SCT, and SDT theories, this study develops a conceptual model of chained mediation that integrates technological, psychological and behavioral variables. This accounts for the distinctiveness of this research. Based on this foundation, this study specifically targeted adolescents aged 6–18 years across the country and used a non-probability sampling approach combining a quota and a non-probability sample to obtain survey data. A total of 1,018 questionnaires were collected, covering over 90% of Chinese provinces. The aim of this study is to examine the effect of generative AI anthropomorphism on exercise motivation in adolescents using these data, based on a chain-mediated model of technology acceptance and self-efficacy.

It is worth noting that the potential risks of AI anthropomorphism cannot be ignored. These risks manifest at the psychological level as subjective discomfort induced by the “uncanny valley” effect (Kopp et al., 2022); at the data security level as concerns over privacy breaches and behavioral surveillance (Chuah et al., 2021); and at the social interaction level as excessive reliance on virtual empathy and the deterioration of real-world social skills (Huang and Rust, 2024). The application of AI anthropomorphism in educational environments, though aimed at increasing learning motivation, poses substantial ethical risks, including algorithmic bias, diminished accountability, and the exacerbation of educational inequity (Klimova et al., 2023). Therefore, while the positive impact of AI anthropomorphism on enhancing the exercise motivation in youth is fully explored, its potential problems require the close attention of all stakeholders.

Notwithstanding these potential risks, this research aims to deliver theoretical and practical contributions. In theory, it aims to broaden the boundaries of digital physical activity by introducing AI anthropomorphism as a critical motivator. In practice, it aims to provide an empirical basis for developing AI-based interventions to increase physical activity in adolescents, to design AI fitness software interfaces and to develop AI-integrated curricula in sports exercise.

## 2 Literature review and hypothesis

### 2.1 Generative AI and its applications across various fields

While traditional AI systems were designed to emulate human intelligent behavior (Huang and Rust, 2024), the core of generative AI lies in its capacity to create novel artifacts (Bordas et al., 2024), which have the learning systems that generate novel text, images, audio, and video content from user inputs (Taeihagh, 2025). The advanced semantic understanding capabilities of generative AI have fostered its anthropomorphic characteristics, driving interactive formats exemplified by “chatbots” (Baudier and de Boissieu, 2025). Nowadays, AI-powered anthropomorphic chatbots are widely used in various fields, for instance, tourism, hospitality management, marketing, and education.

Numerous studies increasingly demonstrate that AI anthropomorphism is an effective measure for enhancing user engagement, fostering acceptance, and stimulating motivation across a broad array of service domains. For instance, in tourism and hospitality management, anthropomorphic robots have had a significant impact on service quality and competitiveness (Saputra et al., 2024); in healthcare, AI’s human-like qualities have fostered patient acceptance by demonstrating empathy (Li W. et al., 2024); in marketing, anthropomorphic AI is associated with products’ online reputation (Fatima et al., 2024); in education, AI has been integrated into higher education (Soliman et al., 2024) and K-12 curricula, improving learning motivation and performance by addressing diverse educational needs (Xia et al., 2022). These research indicates that AI anthropomorphism will become increasingly integrated into various sectors and serve as an important facilitator in human life (Blut et al., 2021).

In youth sports, AI anthropomorphism has had a twofold impact. Evidence shows that it has considerable potential to reinforce digital health interventions and increase levels of physical activity (Wang et al., 2025). Conversely, scholars point out that despite these advances, the indispensable role of human expertise remains (Saraç et al., 2025). Furthermore, they caution that potential negative effects deserve attention, even though adolescents may have some insight into the ethics of artificial intelligence (Huang et al., 2024). These divergent outcomes point to a complex underlying mechanism. To disentangle these underlying mechanisms, this study narrows its focus to exercise motivation to explore this relationship.

### 2.2 Theoretical foundations

#### 2.2.1 Unified theory of acceptance and use of technology (UTAUT)

Developed by Venkatesh et al. (2012), the UTAUT 2 model extends the original UTAUT 1 (Venkatesh et al., 2003) by complementing its four core constructs—performance expectancy, effort expectancy, social influence, and facilitating conditions—with three additional predictors: hedonic motivation, price value, and habit. Given the accelerating pace of innovation in artificial intelligence, UTAUT 2 has attracted considerable scholarly interest for its applicability and efficacy in explaining technology adoption behaviors. For instance, an educational study demonstrated that users’ perceived validation significantly improve their satisfaction and subsequent willingness to continue using AI chatbots. In the specific context of AI anthropomorphism, its functional features—such as the provision of personalized and autonomous learning methods (Bays et al., 2024) and immersive exercise guidance (Wang et al., 2025)—correspond to constructs in the UTAUT2, including effort expectancy and hedonic motivation. This correspondence thereby informs the theoretical framework for investigating how AI anthropomorphism is associated with exercise motivation.

#### 2.2.2 Social cognitive theory (SCT)

Social cognitive theory (SCT), proposed by Bandura, posits a theoretical framework of triadic interaction among individuals, environments, and behaviors (Bandura, 1989). Within the framework, individual factors are conceptualized as endogenous determinants such as self-efficacy, attitudes, and expectations; environmental factors represent exogenous conditions, encompassing both physical and social contexts; and behavioral factors refer to the observable actions or responses enacted by the individual. Nowadays, the increasing prominence of AI anthropomorphism, which serves as a novel environmental factor within SCT, amplifies the theory’s explanatory capacity. Existing research substantiates that AI anthropomorphism is significantly associated with student learning outcomes through factors such as self-efficacy, moral fairness, and creativity (Shahzad et al., 2025). Therefore, situating AI anthropomorphism within the environmental component of SCT offers a powerful lens for investigating its relationship with adolescents’ self-efficacy and exercise motivation.

#### 2.2.3 Self-determination theory (SDT)

The self-determination theory (SDT), advanced by Deci and Ryan (1985), postulates that an individual’s intrinsic motivation is organized around the satisfaction of three innate needs: competence, autonomy, and relatedness. Among these, competence refers to an individual’s perception of self-efficacy in completing a task; autonomy primarily reflects the degree of freedom in decision-making; relatedness primarily reflects the perceived capacity to form emotional bonds with others. These three needs serve as prerequisites for intrinsic motivation, thereby exerting an indirect link to actual behavior. The anthropomorphic design of artificial intelligence demonstrably facilitates the activation of human intrinsic motivation. For instance, SDT-based educational research indicates that autonomy of AI is positively associated with the intention of university students to use it (Soliman et al., 2025); conversely, overly utilitarian AI detrimentally affects learning outcomes (Hsia et al., 2025). Therefore, the SDT framework could provide theoretical support for investigating the relationship between AI anthropomorphism and technology acceptance, self-efficacy and exercise motivation in this study.

#### 2.2.4 A chain-mediated model: integrating UTAUT, SCT, and SDT

The key feature of chained mediators is their ability to reveal the full causal chain from independent to dependent variables, while breaking down the specific impacts of each path. Therefore, this study constructs a chained mediation model with technology acceptance and self-efficacy as mediating variables based on theoretical integration, aiming to reveal the causal progression of AI anthropomorphism from technological characteristics through internal psychological mechanisms to behavioral levels.

### 2.3 Research hypothesis

#### 2.3.1 AI Anthropomorphism and adolescents’ exercise motivation

According to self-determination theory (SDT), individuals possess distinct psychological needs and motivational types, which are associated with external environmental factors (Xia et al., 2022). Given that generative AI can provide real-time guidance for exercise motivation (Luna and Denham, 2022), its anthropomorphic design may significantly improve adolescents’ exercise motivation by reducing technological barriers and enhancing interaction willingness. In the field of youth sports research, the emergence of AI anthropomorphism driven by rapid technological iteration and advancement has become a novel and significant precedent. First, the design of AI anthropomorphism stimulates adolescents’ emotional experiences through the mediating roles of perceived usefulness and consumption willingness (Zhang and Wang, 2023). This heightened engagement fosters a critical stance, leading adolescents to persistently question the nature of their involvement in athletic activities. Second, the interpersonal interaction facilitated by anthropomorphic features provides sustained motivation for adolescents to participate in sports. This novel and interactive approach empowers adolescents to continually overcome the issues encountered during exercise (Lakicevic et al., 2020). Therefore, the following hypothesis is proposed.

H1: AI anthropomorphism is positively associated with adolescents’ exercise motivation.

#### 2.3.2 The mediating role of technology acceptance between AI anthropomorphism and exercise motivation

According to Unified theory of acceptance and use of technology (UTAUT 2), hedonic motivation, price value, and habit are associated with users’ acceptance of technology. Notably, AI anthropomorphism can precisely provide the necessary conditional support for the emergence of these three factor. However, most existing studies on sports motivation focus on teaching methods or learning performance, while empirical research exploring the impact of AI technology acceptance on adolescents’ learning motivation remains limited (Fan and Ye, 2022; Hsu and Rowland-Goldsmith, 2021). A significant positive association between AI anthropomorphism and motivation for physical activity can be hypothesized based on many existing studies. For example, the real-world effectiveness of AI in competitive sports highlights the value of AI for correcting movements (Liu et al., 2023), predicting exercise risk (Musat et al., 2024), and providing nutritional programs (Puce et al., 2024). Additionally, studies on other technologies, such as fitness trackers and sports bracelets (Wang et al., 2022) and VR (Sun and Ke, 2025), have demonstrated that technology acceptance is associated with motivation in sports contexts. Therefore, the following hypothesis is proposed.

H2: Technology acceptance mediates the relationship between AI anthropomorphism and exercise motivation.

#### 2.3.3 The mediating role of self-efficacy between AI anthropomorphism and exercise motivation

As a well-established framework, Social cognitive theory (SCT) provides a theoretical foundation for the study of motivation (Schunk and DiBenedetto, 2020), in which self-efficacy plays a central role. Existing empirical studies have shown that self-efficacy has a significant positive effect on exercise motivation (Yu and Song, 2022; Zhang et al., 2024). Given the critical role of self-efficacy in adolescent sport behavior, identifying its associations has become an important direction for expanding theoretical boundaries and guiding practice. The increased anthropomorphism of AI improves consumer acceptance and fosters positive emotions (Song et al., 2023), which can significantly improve users’ willingness to interact with it (Mehmood et al., 2024). Previous research also has demonstrated that the utilization of digital technology is positively associated participation in sports training (Qian and Kim, 2025). Based on the above reasoning, we infer that the AI anthropomorphism has a significant positive impact on adolescents’ exercise motivation. Therefore, the following hypothesis is proposed.

H3: self-efficacy mediates the relationship between AI anthropomorphism and exercise motivation.

#### 2.3.4 Technology acceptance and self-efficacy as chain mediators between AI anthropomorphism and exercise motivation

When adolescents perceive AI anthropomorphism as a supportive tool for achieving their initial physical exercise goals, they are prone to sustained engagement with the AI, aiming for amplified exercise effectiveness and a consequential implicit enhancement of their self-efficacy. According to social cognitive theory (SCT), self-efficacy, as an individual’s subjective assessment of their capabilities, mediates the relationship between the environment and behavior (Lin et al., 2024). Specifically, higher levels of self-efficacy are associated with intentions among adolescents to participate in sports (Su and Liu, 2025). This indicates that AI anthropomorphism is associated with adolescents’ self-efficacy through interactions, thereby improving their physical exercise motivation. However, few studies have explored how technology acceptance and self-efficacy simultaneously mediate the impact of AI anthropomorphism on exercise motivation. Therefore, the following hypothesis is proposed.

H4: Technology acceptance and self-efficacy sequentially mediate the relationship between AI anthropomorphism and exercise motivation.

## 3 Materials and methods

### 3.1 Participants

The survey instrument for this study consisted of three primary components: an introductory statement, demographic information items, and multi-item scales measuring four latent constructs. Specifically, the scale section incorporated measures of AI Anthropomorphism (8 items), Technology Acceptance (6 items), Self-Efficacy (6 items), and Exercise Motivation (7 items), yielding a total of 27 items. All scales utilized a five-point Likert-type response format, ranging from 1 (strongly disagree) to 5 (strongly agree).

About the minimum sample size, this study following the recommendation by Bentler and Chou (1987) that sample size should be 5–15 times the number of observed variables. Given the complexity of the measurement model, a stringent sampling criterion of 15 participants per estimated parameter was adopted, yielding a calculated minimum required sample size of 405. This approach to determining sample size follows the guidelines established by Hair et al. (2009).

This study recruited a non-probability online sample of 1,072 adolescents (aged 6–18) from 29 provincial divisions in China (over 85% national coverage) via the Question-Star platform. After excluding 54 invalid questionnaires based on reverse-scored items and completion time (< 60 seconds), 1,018 valid responses were retained (94.9% effective rate). Although the non-probability sampling approach limits generalizability, the resulting sample (*N* = 1,018) is deemed sufficient for structural equation modeling analysis using AMOS, aligning with the main aim of exploring the relationships among four variables.

### 3.2 Measures

#### 3.2.1 AI anthropomorphism

This AI anthropomorphism scale by Shen et al. (2024) measures adolescents’ perceived level of AI anthropomorphism. It consists of 16 items, covering personality, empathy, and mind. This scale employs the Likert five-point scale. The AI anthropomorphism scale in this paper has been proven to be highly reliable, as demonstrated by Cronbach’s α of 0.918 and a satisfactory Corrected Item-Total Correlation (CITC). Regarding construct validity, confirmatory factor analysis showed excellent model fit indices: χ^2^/df = 0.746, RMR = 0.011, CFI = 0.999, GFI = 0.996, TLI = 0.999, RMSEA = 0.001, all meeting established criteria for good fit.

#### 3.2.2 Technology acceptance

This study used the Generative Artificial Intelligence Acceptance (GAIA) scale developed by Yilmaz et al. (2024), which consists of 20 items representing four dimensions (Performance expectancy, effort expectancy, facilitating conditions, social influence). A five-point Likert scale (1 = Strongly disagree, 5 = Strongly agree) was employed for each item to measure the technology acceptance. The technology acceptance scale in this paper has been proven to be highly reliable, as demonstrated by Cronbach’s alpha of 0.907 and a satisfactory CITC. Regarding construct validity, confirmatory factor analysis showed excellent model fit indices: χ^2^/df = 1.179, RMR = 0.012, CFI = 0.999, GFI = 0.997, TLI = 0.999, RMSEA = 0.013, all meeting established criteria for good fit.

#### 3.2.3 Self-efficacy

This study used the General Self-Efficacy Scale (GSES), developed by Schwarzer and Jerusalem (1995), to evaluate adolescents’ confidence in completing physical activities. The Chinese version, translated and revised by Zeng et al. (2022), consists of 10 items, adopting a four-point Likert scale (1 = completely incorrect, 4 = completely correct). The self-efficacy scale in this paper has been proven to be highly reliable, as demonstrated by Cronbach’s alpha of 0.902 and a satisfactory CITC. Regarding construct validity, confirmatory factor analysis showed excellent model fit indices: χ^2^/df = 1.797, RMR = 0.015, CFI = 0.999, GFI = 0.995, TLI = 0.996, RMSEA = 0.028, all meeting established criteria for good fit.

#### 3.2.4 Exercise motivation

Exercise motivation was assessed using the Chinese version of the “Motives for Physical Activities Measure-Revised Scale” (MPAM-R) developed by Nader et al. (2021). This scale contains 15 items across five dimensions: social motivation, appearance motivation, ability motivation, health motivation, and enjoyment motivation. The items were rated using a five-point scale (1 = not conforming at all, 5 = conforming completely). This scale has been validated effectively in studies on students’ participation motivation (Sun and Ke, 2025). The exercise motivation scale in this paper has been proven to be highly reliable, as demonstrated by Cronbach’s alpha of 0.915 and a satisfactory CITC. Regarding construct validity, confirmatory factor analysis showed excellent model fit indices: χ^2^/df = 1.580, RMR = 0.015, CFI = 0.998, GFI = 0.994, TLI = 0.997, RMSEA = 0.024, all meeting established criteria for good fit.

## 4 Results

### 4.1 Subject demographic data

In order to establish a foundational understanding of the study participants, descriptive statistics were utilized to summarize the demographic profile of the valid sample (*N* = 1,018). Among the valid surveyed participants, 483 were male, accounting for 47.4%, while 535 were female, comprising 52.6%. The respondents were aged between 6 and 18 years old, with the 12–15 age group accounting for the highest proportion. Regarding school-age periods, 19.9% were preschool children, 40.1% were primary school students, 33.4% were junior high school students, and 17.9% were senior high school students. The number of sports projects mastered by the respondents varies, and the proportion of those who master three sports is the highest. In terms of AI usage frequency, the proportion of people who use AI 1–2 times a week is relatively high. The proportions of these two frequencies are 36.3% and 26.7%, respectively, with a total proportion of 63%. The demographic information is shown in Table 1.

**TABLE 1:** Descriptive statistics of the sample.

Demographic variables	Category	Frequency	Percentage
Gender	Male	483	47.4
	Female	535	52.6
Age	(6–11)	270	26.5
	(12–15)	408	40.1
	(16–18)	340	33.4
School-age period	Preschool children	203	19.9
	Primary school	408	40.1
	Junior high school	340	33.4
	Senior high school	182	17.9
Number of sports	1	67	6.6
	2	203	19.9
	3	408	40.1
	4	340	33.4
	>5	182	17.9
Al usage frequency	≤1	370	36.3
	2	272	26.7
	3	128	12.6
	4	66	6.5
	>5	52	5.1

### 4.2 Examination of bias

#### 4.2.1 Self-report bias test

In order to reduce the bias in self-reported data, the study carried out the following measures during the questionnaire design phase. First, we guaranteed anonymity and informed participants that the data would serve solely for academic research, thereby mitigating evaluation apprehension and social desirability. Second, this study reworded items from established scales into a more colloquial style, tailoring them for easier comprehension by adolescents, particularly those in younger age groups. Third, this study embedded a reverse-scored item (e.g., “I believe AI cannot provide effective guidance for sports participation”) within the technology acceptance dimension. All questionnaires in this study were completed voluntarily with informed consent.

#### 4.2.2 Common method bias test

This study implemented two distinct analytical approaches to assess potential to check for common method bias: Harman’s single-factor test and a comparison of competing measurement models via confirmatory factor analysis (Podsakoff et al., 2003). First, a single-factor Harman test performed on all items extracted four factors collectively accounting for 66.154%of the total variance, with the first factor explaining only 37.3% of the variance (below the 50% threshold). This indicates that common method bias is not pervasive in the data. Second, the results of the competitive factor model analysis indicate that the theoretical four-factor model showed an excellent fit to the data (χ^2^/df = 1.356, RMSEA = 0.019, RMR = 0.027, CFI = 0.993, TLI = 0.992, IFI = 0.993). Conversely, a single-factor model exhibited a significantly poor fit (χ^2^/df = 23.898, RMSEA = 0.150, RMR = 0.181, CFI = 0.548, TLI = 0.510, IFI = 0.549). Overall findings suggest that there are no severe common method bias issues in the present study.

### 4.3 Assessment of reliability and validity

This study used SPSS 27.0 to analyze the reliability and validity of variables. The study showed that the Cronbach’s α coefficients of all variables exceeded 0.9. Specifically, the coefficient for AI anthropomorphism was 0.918, for technology acceptance was 0.907, for self-efficacy was 0.902, and for exercise motivation was 0.915. The KMO values is 0.958, and Bartlett’s test of sphericity was significant (*p* < 0.01).

As a widely used relational technique, correlation analysis in this study examines the linear relationship between two variables. Table 2 presented the correlation coefficients and the discriminant validity. The average variance extracted (AVE) ranged from 0.580 to 0.599 with composite reliability (CR) all exceeding 0.90. The composite reliabilities (CR) of AI anthropomorphism, technology acceptance, self-efficacy, and exercise motivation were 0.92, 0.90, 0.90, and 0.92, respectively, while AVE were 0.5803, 0.5598, 0.5989, and 0.596, respectively, all meeting the reliability and validity criteria. The findings demonstrate good convergent validity of the scale.

**TABLE 2:** Descriptive statistics and bivariate correlations (*n* = 1,018).

Variables	M	SD	1	2	3	4
AI anthropomorphism	3.396	0.919	1			
Technology acceptance	3.352	0.984	0.438***	1		
Self-efficacy	3.375	0.957	0.397***	0.433***	1	
Exercise motivation	3.327	0.953	0.390***	0.437***	428***	1

***p < 0.001. M.mean; SD, standard deviation.

Confirmatory factor analysis (CFA) confirms the factor structure of observed variables and provides tools for measuring model validity in SEM (Marsh et al., 2020). The results showed that the fitting of each model is good, and scale validity is good (Table 3).

**TABLE 3:** Validation factor model fit.

Fit index	x^2^/df < 3.000	CFI	TLI	GFI	NFI > 0.900	IFI	RMR < 0.050	RMSEA < 0.050
Reference value	<5.000	>0.900	>0.900	>0.900	>0.9004	>0.900	<0.080	<0.080
Test value	1.354	0.993	0.992	0.968	0.974	0.993	0.027	0.019

### 4.4 Hypothesis testing

This study utilized linear regression analysis to explore the associations between AI anthropomorphism integrated with technology acceptance, self-efficacy, and exercise motivation. Based on the hypothesized connections among these variables, four regression models were constructed. The research findings were as follows in Table 4.

**TABLE 4:** Regression analysis of AI anthropomorphism cooperated with technology acceptance, self-efficacy, and exercise motivation.

Predictor variable	Outcome variable	*R*	*R* ^2^	*F*	β	*t*	BootLLCI	BootULCI
**Equation 1**								
Al anthropomorphism	Technology acceptance	0.4362	0.1903	79.4296	0.4648	15.3036	0.4052	0.5245
**Equation 2**								
Al anthropomorphis	Self-efficacy	0.4911	0.2412	80.5094	0.2647	8.3363	0.2024	0.3270
Technology acceptance					0.3120	10.5443	0.2539	0.3700
**Equation 3**								
Al anthropomorphism	Exercise motivation	0.5367	0.2881	81.9007	0.1877	5.9248	0.1255	0.2499
Technology acceptance					0.2415	8.0263	0.1824	0.3005
Self-efficacy					0.2446	8.0658	0.1851	0.3041

All four models yielded significant results (*p* < 0.001), as shown in Table 5. Equation 1 revealed a notably positive relationship between AI anthropomorphism and exercise motivation. Equation 2 revealed that AI anthropomorphism, together with technology acceptance, was positively correlated with self-efficacy. Equation 3 showed that AI anthropomorphism, together with technology acceptance and self-efficacy, had significantly positive effects on exercise motivation.

**TABLE 5:** Mediation effect results.

Path	Effect value	SE	*P*-value	Bias-corrected 95% CI		Mediation percentage (%)
				Lower	Upper	
Total effect	0.4002	0.0301	*p* < 0.001	0.3412	0.4592	100.0
Direct effect	0.1877	0.0317	*p* < 0.001	0.1255	0.2499	46.9
Indirect effect	0.2044	0.0191	*p* < 0.001	0.1765	0.2505	51.1
Ind I	0.108	0.0156	*p* < 0.001	0.0828	0.1431	27.0
Ind 2	0.0623	0.0110	*p* < 0.001	0.0448	0.0871	15.6
Ind 3	0.0341	0.0060	*p* < 0.001	0.0248	0.0477	8.5

**FIGURE 1:**
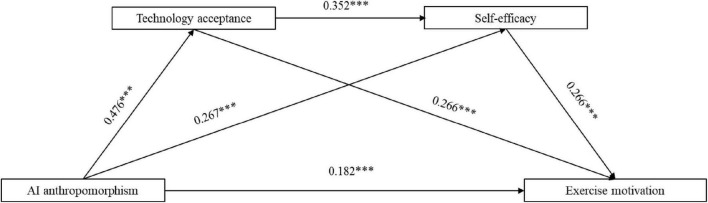
The chain mediating model of AI anthropomorphism on exercise motivation. ***Indicates *p* < 0.001.

As shown in Table 5, a positive relationship exists between AI anthropomorphism and exercise motivation [path coefficient = 0.1877, 95% CI (0.1255, 0.2499), *p* < 0.001], supporting hypothesis H1. There is a significant mediating effect of technology acceptance on the relationship between AI anthropomorphism and exercise motivation, with an effect value of 0.108 and a 95% confidence interval of (0.0828, 0.1432), and the *p*-value is < 0.001, which supports hypothesis H2. There is a significant mediating effect of self-efficacy on the relationship between AI anthropomorphism and exercise motivation, with an effect value of 0.0623 and a 95% confidence interval of (0.0448, 0.0871), and the *p*-value is < 0.001, which supports hypothesis H3. There is a significant chain mediating effect of technology acceptance and self-efficacy on the relationship between AI anthropomorphism and exercise motivation, with an effect value of 0.0341 and a 95% confidence interval of (0.0248, 0.0477), and the *p*-value is < 0.001, which supports hypothesis H4. The research findings informed the development of the chain mediation model, which is visualized in Figure 1.

### 4.5 Multi-group analysis of indirect effects across age groups

#### 4.5.1 Tests of configuration invariance

To examine the applicability of the hypothesized serial mediation model across the three age groups, a multi-group confirmatory factor analysis (MG-CFA) was conducted using AMOS 24. The sample was partitioned into three subgroups (6–11, 12–15, and 16–18 years) for this analysis. Initially, a configural invariance model (the baseline model) was specified, in which the factor structure was held constant across groups while allowing all parameters to be freely estimated. The results demonstrated that this unconstrained baseline model achieved an acceptable level of model fit across all three age groups (as presented in Table 6), with all key indices meeting the conventional thresholds for good fit. This establishment of configural invariance indicates that the fundamental structure of the model is equivalent across groups, thereby justifying subsequent, more restrictive tests of measurement and structural invariance.

**TABLE 6:** Goodness-of-fit indices for the configural invariance by age group.

Age group	X^2^	df	x^2^/df	RMSEA	CFI	TLI	RMR
Model 1: (6–11)	1291.526	1075	1.201	0.014	0.987	0.987	0.0434
Model 2: (12–15)	1470.391	1075	1.368	0.018	0.979	0.98	0.0378
Model 3: (16–18)	1255.425	1075	1.168	0.012	0.99	0.99	0.0338

#### 4.5.2 Tests of structural path invariance

Based on the established configuration invariance of the base model (model 1), further tests were conducted to assess measurement invariance. First, the metric invariance model (model 2) was defined by limiting all factor loads to be the same for all three age groups. The results of the comparison of the two models are as follows: Δx^2^ = 34.74, Δdf = 44, *p* = 0.84, ΔCFI = −0.0002 (< 0.01), ΔRMSEA = −0.001 (< 0.015), *p* > 0.05. It can be concluded that the measurement of latent variables exhibits invariance across different age groups.

After confirming the invariance of measurements, this study examined whether there were age differences in the chained mediation model. A structural weighting model (Model 3) was developed, in which the coefficients of the structural paths were constrained to be equal across all three age groups. A comparison of model 3 with model 2 resulted in the following results: Δχ^2^ = 6.075, Δdf = 12, *p* = 0.912, ΔCFI = 0, ΔRMSEA = 0. Based on the criteria established by Cheung and Rensvold (2002), it can be concluded that structural path invariance holds, meaning that the path coefficients of the chained mediation model proposed in this study do not exhibit significant differences across different age groups. Results of the structural path invariance tests are shown in Table 7.

**TABLE 7:** Measurement invariance tests across age groups.

Age group	X^2^	df	x^2^/df	RMSEA	CFI	Ax^2^	Adf	Ax^2^/df	ARMSEA	ACFI	*P*-value
Unconstrained	1199.26	957	1.253	0.016	0.985	–	–	–	–	–	–
Measurement weights	1234	1001	1.233	0.015	0.986	34.74	44	−0.02	−0.001	0.001	0.84
Structural weights	1240.075	1013	1.224	0.015	0.986	6.075	12	−0.009	0	0	0.912

#### 4.5.3 Comparison of specific path differences

Although overall structural invariance was established, descriptive comparisons of specific paths were conducted to provide more detailed information. As shown in Table 8, all core path factors were significant in all three age groups and had similar magnitude. In addition, we examined the matrix of critical ratios and confirmed that for all core routes, the absolute critical ratio in the intergroup comparison was below 1.96. These results provide additional parametric support for the conclusion that structural path invariance is maintained, indicating that the chained mediation model is highly stable and consistent across different age ranges.

**TABLE 8:** Standardized path coefficients in the structural model.

Path			Age (6–11)		Age (12–15)		Age (16–18)	
			β	SE	β	SE	β	SE
Technology acceptance	<—	AI anthropomorphism	0.425***	0.076	0.5***	0.059	0.483***	0.058
Self-efficacy	<—	Technology acceptance	0.304***	0.065	0.346***	0.053	0.407***	0.068
Self-efficacy	<—	AI anthropomorphism	0.288***	0.073	0.295***	0.057	0.204***	0.062
Exercise motivation	<—	Self-efficacy	0.206**	0.069	0.279***	0.068	0.308***	0.055
Exercise motivation	<—	AI anthropomorphism	0.205**	0.072	0.157**	0.065	0.202***	0.053
Exercise motivation	<—	Technology acceptance	0.292***	0.065	0.216***	0.061	0.302***	0.06

***Indicates *p* < 0.001,

**indicates *p* < 0.01.

All core paths remained significant across age groups (*p* < 0.01 or *p* < 0.001), affirming broad applicability. Under confirmed overall model invariance, subtle age-related differences in significance levels were observed in specific pathways. The motivational effects of both self-efficacy and AI anthropomorphism reached their highest statistical significance in older age groups. These results indicate that intervention strategies should differentially prioritize self-efficacy and AI anthropomorphism based on the target age group’s developmental stage.

## 5 Discussion

### 5.1 AI anthropomorphism and Exercise motivation

The present study confirms a significant positive relationship between adolescents’ AI anthropomorphism and their exercise motivation in sports, supporting research hypothesis 1. This is closely associated with the capability of AI anthropomorphism in personalized exercise programs (Wang et al., 2025), lower coaching costs (Bays et al., 2024), the role of emotional regulation (Chew, 2022), and its correlations with mental health, among other factors (Qi, 2025). It is noteworthy that Saraç et al. (2025) also highlighted that while AI chatbots have positive effects on exercise, there are certain discrepancies between human trainers and chatbots in terms of key parameter recommendations. However, research by Guo et al. (2025) demonstrates that AI’s judgment capabilities exhibit strong consistency with those of humans. The existence of such contradictory findings may be related to the type of software involved, as suggested by Kuhail et al. (2023), and the early developmental stage of educational AI software (Hwang and Chang, 2023). This further underscores the necessity of research on AI anthropomorphism, with the aim of providing more comprehensive insights and theoretical references for AI software developers. Furthermore, this study can provide additional empirical support for the perspective advanced by Gut et al. (2020), which emphasizes human-centric intervention measures in physical exercise motivation. Thereby, it offers a more systematic theoretical framework for enhancing exercise motivation among adolescents.

### 5.2 The mediating role of technology acceptance

This study confirms that technology acceptance mediates the relationship between AI anthropomorphism and exercise motivation, thereby supporting Research Hypothesis 2. Specifically, a higher degree of AI Anthropomorphism correlated with greater acceptance and willingness among youth. This finding aligns with existing research. Xie et al. (2023) proposed that the AI anthropomorphism can improve human-AI interaction satisfaction. This positive effect is further demonstrated in the context of mobile fitness applications, where it was shown to increase users’ willingness to continue use (Lee and Lin, 2023). Furthermore, technology acceptance positively affects the relative behavior of exercise motivation, and prior studies have confirmed its key role in behavior. For example, drawing on the successes of the virtual reality sports system (Wang et al., 2025) and InPACT at Home (Sapre et al., 2025), we can create a system that supports mental health, encourages exercise, encourages greater levels of physical activity, and develops healthy social relationships.

It is worth noting that, the effectiveness of AI anthropomorphism demonstrates a significant cultural variance. Cross-cultural studies show that East Asian cultural contexts (China, India) show a higher acceptance and a greater preference for interaction with anthropomorphic artificial intelligence than users from European and American cultures (Folk et al., 2025; Fatima et al., 2024). There are, however, some opposing views in the literature. For example, Frank (2024) argues that Chinese users attach less importance to social connections mediated by artificial intelligence than their US and Japanese counterparts, and Liu et al. (2023) notes that users in mainland China place more emphasis on emotional characteristics of artificial intelligence than users in Hong Kong. These findings highlight the contextual complexity of cultural effects on AI anthropomorphism, which demands further investigation across different domains, populations, and regions.

### 5.3 The mediating role of self-efficacy

This study found that the positive association of AI anthropomorphism on exercise motivation could be achieved through both a direct pathway and an indirect pathway mediated by self-efficacy, supporting hypothesis H3. This suggests that the rapid development of information technology has significantly broadened the sources of self-efficacy, reaching deep into the online realm, and this conclusion has been confirmed (Shahzad et al., 2025). For example, AI systems with anthropomorphic features is associated with adolescents’ self-efficacy by providing more approachable, emotionally engaging, and personalized interactive experiences (Cao et al., 2022; Cheng et al., 2022). In contrast to impersonal mechanical feedback, AI anthropomorphism can create more supportive environments and stimulate positive physical practice experiences, thereby strengthening adolescents’ beliefs in their athletic potential (Yang et al., 2025). Adolescents with higher self-efficacy tend to exhibit greater interest in exercise activities (Fu et al., 2023), enabling them to approach the challenges of physical activity with a more positive mindset, which in turn predicts more favorable outcomes (Schunk and DiBenedetto, 2020).

### 5.4 Chain mediation between technology acceptance and self-efficacy

This results indicates that technology acceptance and self-efficacy served as chain mediators between AI anthropomorphism and exercise motivation, confirming that research H4 was established. This finding is consistent with Zhou et al. (2025), who argued that the diverse value functions of AI, including optimizing athletic movement, personalizing training, improving diagnostics, accelerating rehabilitation and anthropomorphic characteristics, is associated with adolescents’ acceptance of generative AI (Zhou et al., 2025). When adolescents tackle the physical activity challenges with AI assistance, they are more inclined to think deeply about the problems they encounter and adopt proactive. As problems are continuously resolved, AI’s timely feedback and encouragement are associated with a higher sense of self-efficacy in physical activities (Zheng et al., 2025). This further illustrates that adolescents’ acceptance of AI mediates the relationship between AI anthropomorphism and self-efficacy. These findings align with those of Shao et al. (2025), whose study additionally suggests that this role relationship is also associated with AI ethics. In summary, the chain-mediating effect underscored the strong positive link between technology acceptance and self-efficacy was the key to positively predicting exercise motivation. The above research findings insight encourages program developers to prioritize adolescents’ preferences in software design to improve students’ perceived usefulness and ease of use regarding AI (Tian et al., 2024).

### 5.5 Practical implication

For schools, three implementations should be adapted to apply anthropomorphic AI for enhancing youth exercise motivation. First, adopt a differentiated appearance for chatbots. Cartoon anthropomorphic chatbots should be promoted to improve emotional interaction among students in lower grades, while priority should be given to professional interfaces to highlight the academic support function of students in higher grades. Secondly, AI courses should be regularly offered, taught by specialists in AI ethics and security, to cultivate students’ skills for critical use. At the same time, AI chatbots should be deployed in public spaces, like gymnasiums, to facilitate students’ access to equipment. Third, governance mechanisms can be strengthened by restricting non-academic content access on the school’s LAN, thereby minimizing the negative impact of chatbots as much as possible.

For coaches, three strategies can be used to leverage AI anthropomorphism. First of all, it is beneficial to actively utilize AI’s inherent strengths in data processing to improve the effectiveness of human–AI collaborative learning. Furthermore, coaches should improve students’ acceptance and application frequency of AI tools by demonstrating human-AI dialogue techniques in detail during instruction, supplemented by motivational strategies such as point-based ranking systems. Finally, coaches should pay more attention to the students who are not particularly interested in physical exercise or find the related technical movements challenging.

For software engineers, three steps should be implemented to optimize the intervention effect of AI anthropomorphism on youth exercise motivation. First, the dialogue strategies should leverage motivational and relaxed language to increase the AI chatbot’s empathic capacity, thereby directly improving the youth’s acceptance. Second, Second, the system should automatically trigger a safety warning, prompting students to seek professional guidance when they inquire about highly complex or potentially hazardous activities. Third, this study recommends that developers develop learning-oriented conversational agents that can automatically curate learning content using specific filtering mechanisms embedded in the system architecture.

## 6 Conclusion

This study confirmed a significant predictive relationship between AI anthropomorphism and adolescents’ physical exercise motivation, revealing a chain mediation effect through technological acceptance and self-efficacy. Specifically, AI anthropomorphism is associated with higher acceptance of intelligent technology, subsequently strengthening their self-efficacy in physical exercise, and ultimately stimulating exercise motivation. These findings have both theoretical and practical significance. At the theoretical aspects, the significance of this study manifests in two dimensions. First, it provides crucial empirical evidence and theoretical reference for interdisciplinary research at the intersection of AI and sports psychology. Second, by examining the specific technological feature of AI anthropomorphism, this study not only expands the boundaries of environmental factors within social cognitive theory (SCT) but also injects new theoretical insights into integrating the Unified theory of acceptance and use of technology (UTAUT) for emerging technological contexts. This significantly improve the explanatory power and universality of relevant theoretical models. At the practical aspects, they provide a digital solution for improving adolescents’ physical activity levels, help adolescents develop exercise habits, and offer optimization ideas for schools, training institutions and other organizations to carry out adolescent sports work, so as to promote the in-depth integration of AI technology and adolescent sports education.

## 7 Limitations and directions for future research

This study is subject to three primary limitations. First, the sample representation and cultural generalizability are constrained. The use of an non-probability sampling method with all participants drawn from the Chinese student population leads to a limited diversity of cultural and demographic composition. Consequently, the external validity of the findings is limited and the results should be interpreted as provisional and subject to change. Second, the research design limits causal interpretation. As a cross-sectional study, it is only possible to identify associations between variables at one time point. The absence of longitudinal data prevents definitive conclusions on the direction and causation of the relationships examined. Thirdly, technology acceptance scale pose a potential constraint due to their status as novel tools. Unlike the widely-validated classical instruments, their long-term reliability, validity and population-wide applicability await further empirical validation.

To advance the study of this topic, future research should systematically explore the following two dimensions. First, methodologically diversified approaches are needed to elucidate the role of AI anthropomorphism on physical activity motivation. For example, qualitative methods should be employed to explore the users’ subjective perception of Anthropomorphic AI tools; experimental studies are warranted to investigate how AI anthropomorphism influence on actual behavioral engagement; longitudinal analyses would help establish temporal precedence and clarify the causal pathways between AI anthropomorphism and exercise motivation. Second, it is imperative for future studies to prioritize cross-cultural comparisons that systematically test for variations in the effects of AI anthropomorphism. Specifically, this requires cross-cultural comparisons (e.g., East Asia versus West, urban versus rural settings) to formally integrate cultural factors as key moderators in the theoretical models of technological uptake in physical activity.

## Data Availability

The raw data supporting the conclusions of this article will be made available by the authors, without undue reservation.
